# Recruitment to a large scale randomised controlled clinical trial in primary care: the Helicobacter Eradication Aspirin Trial (HEAT)

**DOI:** 10.1186/s13063-022-06054-w

**Published:** 2022-02-14

**Authors:** Diane J. Stevenson, Anthony J. Avery, Carol Coupland, F. D. Richard Hobbs, Denise Kendrick, Michael V. Moore, Clive Morris, Greg P. Rubin, Murray D. Smith, Christopher J. Hawkey, Jennifer S. Dumbleton

**Affiliations:** 1 STAR (Simple Trials for Academic Research) Unit, Nottingham Digestive Diseases Centre, University of Nottingham, Nottingham, UK; 2 Division of Primary Care, University of Nottingham, Nottingham, UK; 3 Nuffield Department of Primary Care Health Sciences, University of Oxford, Oxford, UK; 4 Primary Care Population Sciences and Medical Education, University of Southampton, Southampton, UK; 5 TCR (Nottingham) Ltd., Langley Mill, Nottingham, UK; 6 Institute of Population Health Sciences, Newcastle University, Newcastle-upon-Tyne, UK; 7 School of Health & Social Care, University of Lincoln, Lincoln, UK

**Keywords:** Clinical trial, Recruitment, Primary care, Clinical research networks, Demographics, *H. pylori*, Aspirin, Ulcer bleeding

## Abstract

**Background:**

The Helicobacter Eradication Aspirin Trial (HEAT) is a multicentre, double blind, randomised controlled trial investigating whether *Helicobacter (H.) pylori* eradication reduces hospitalisation for peptic ulcer bleeding. Recruited participants were aged 60 and over and taking aspirin (≤325 mg daily) for at least four months prior to consent. Based on results of a pilot study, a sample size calculation predicted 6600 *H. pylori-*positive randomised participants would be required, from 33,000 volunteers, recruited from 170,000 invited patients. Methodology was therefore designed for recruitment of large numbers of patients from primary care using a novel electronic search tool, automated mail-out and electronic follow-up. Recruitment started in 2012 and completed in 2017.

**Methods:**

All participants were recruited from GP practices, with assistance from the UK Clinical Research Network (UKCRN). *H. pylori-*positive participants were randomised to one week of eradication treatment or placebo. Recruitment was managed using a bespoke web-based database that communicated directly with a programmed search tool downloaded at participating practices. The primary endpoint is hospitalisation due to peptic ulcer bleeding. The trial will end when 87 adjudicated events have occurred, identified from searches of GP databases, review of secondary care admission data and mortality data, and reported events from randomised participants and GPs.

**Results:**

HEAT has recruited participants from 1208 GP practices across the UK. Of the 188,875 invitation letters sent, 38,771 returned expressions of interest. Of these, 30,166 patients were consented to the trial, of whom 5355 *H. pylori*-positive participants (17.8% of those consented) were randomised.

Mean age at consent was 73.1 ± 6.9 (SD) years and 72.2% of participants were male. Of the randomised (*H. pylori*-positive) participants, 531 have died (as of 17 Sep 2020); none of the deaths was due to trial treatment.

**Conclusion:**

The HEAT trial methodology has demonstrated that recruitment of large numbers of patients from primary care is attainable, with the assistance of the UKCRN, and could be applied to other clinical outcomes studies.

**Trial registration:**

ClinicalTrials.gov; registration number NCT01506986. Registered on 10 Jan 2012.

**Supplementary Information:**

The online version contains supplementary material available at 10.1186/s13063-022-06054-w.

## Background

The Helicobacter Eradication Aspirin Trial (HEAT) is a National Institute of Health Research (NIHR)-funded double-blind placebo-controlled randomised trial designed to investigate the hypothesis that *H. pylori* eradication will reduce the incidence of ulcer bleeding in patients taking aspirin [1]. In England in 2017/2018 and 2018/2019, there were over 25,000 hospital admissions for gastric/duodenal ulcers [2], from which in 2017, there were 1866 deaths [3]. If successful, the HEAT trial could improve health outcomes by increasing patient safety and reducing hospital admissions.

Although *H. pylori* infection is becoming less prevalent in the developed world, the level of infection is often higher in economically disadvantaged communities, some ethnic groups and migrants [4]. A study measuring active infection with *H. pylori* in the general population of England and Wales suggested that prevalence was related to decade of birth, and increased from 4.3% in people born in the 1980s to 30% in those born before 1940 [5]. The same authors also demonstrated regional differences in prevalence, which was highest in London and the North of England. They hypothesised that this may be related to household overcrowding and social deprivation.

The HEAT trial has three objectives:

1. To test the hypothesis that a one-week course of *H. pylori* eradication therapy in patients aged 60 or over taking aspirin ≤325 mg daily reduces the incidence of subsequent peptic ulcer bleeding
2. To test the hypothesis that the intervention is cost-effective
3. To establish an inexpensive methodology for performing large simple outcomes trials in primary care

Trial design was informed by an earlier pilot study in which 37% of those invited volunteered to take part, and of those 22% were *H. pylori*-positive. Using these figures, it was estimated that a full trial would need 6600 randomised (*H. pylori*-positive) participants from approximately 33,000 consented patients, in order to detect a hazard ratio of 0.5 for peptic ulcer bleeds comparing the intervention with the control arm, with a 5% two-sided significance level and 90% power.

In order to achieve the required number of participants, the United Kingdom Clinical Research Network (UKCRN) was approached to aid recruitment of GP practices and patients. In each of the four UK nations, clinical research networks have been established whose aim is to provide the infrastructure to support clinical research studies [6]. In England, this infrastructure is organised through the NIHR CRN that is composed of 15 local CRNs that cover all the Clinical Commissioning Groups (CCGs) and deliver research across 30 clinical specialities, one of which is primary care. The Scottish CRN covers 14 Local Health Boards (LHB) and has 7 topic-specific research networks including primary care. Wales has a clinical research infrastructure provided through Health and Care Research Wales covering 7 LHBs, and the Northern Ireland CRN covers nine areas of interest across 5 Health & Social Care Trusts (HSCT) with a coordinating centre based in Belfast.

Patient recruitment to HEAT has been solely from GP practices. Recruitment to clinical trials can be difficult, particularly in primary care, where factors related to the protocol, the clinical setting or the research setting can all play a part [7]. With this in mind, the trial was designed to provide the lowest workload possible for participating GP practices, and minimal face-to-face visits for patients. Practices were provided with a programmed search tool (HEAT Toolkit) that identified eligible patients, and all invitation letters were sent using a highly secure automated online mail management system (Docmail [8]).

One of the principal aims of the HEAT trial was to streamline the methodology of large-scale clinical trials performed in primary care, minimising the impact on GP practices and their patients. This paper describes the methods used and assesses their success in recruitment across the UK.

## Methods

GP practices were recruited through local CRN research facilitators and from previous contacts who had taken part in other studies managed by the HEAT team. Lead GPs at each practice were designated as Study Site Coordinators (SSCs) rather than Principal Investigators and had no responsibility for obtaining regulatory approvals. No recruitment targets were set, although practices with a list size of 5000 or more were preferred. Patient recruitment was nurse-led rather than GP-led, which meant that the trial could provide basic Good Clinical Practice training for the SSCs which occupied less of their time than full NIHR training.

Full details of the methodology have been previously published [1]. Briefly, eligible patients were identified by an electronic search tool downloaded at participating GP practices (HEAT Toolkit). The Toolkit selects eligible patients by using a set of MIQUEST queries. MIQUEST (Morbidity Information Query and Export Syntax [9]) is a specification that utilises a series of queries written in Health Query Language and is also a method of receiving the responses to the queries and distributing them. It is implemented in all GP clinical systems. Using such a system ensured that all practices performed a detailed, identical search that provided an accurate list of patients, each with a unique screening number, which required minimal checking by the SSC.

Eligible patients were ≥ 60 years old, currently on long-term aspirin (≤ 325 mg daily for at least 4 months) and not on anti-ulcer therapy, oral non-steroidal anti-inflammatory drugs or any medication with a clinically significant interaction with the *H. pylori* eradication treatment. Patient invitations were sent out via Docmail [8], an online mailing system approved by Connecting for Health that uses the highest strength encryption for data transfer and the highest level of physical and IT security for mail processing. Practices were simply required to login to the HEAT account on the Docmail website and upload the list of eligible patients produced by the HEAT Toolkit. Having a dedicated HEAT Docmail account enabled complete version control of trial documents posted out to the patients.

Patient recruitment was performed principally by CRN research nurses, but also by research-active GP practice nurses and four dedicated trial research nurses based in the regional centres. Interested patients were seen once at their local GP practice for consent and a *H. pylori* breath test. During the consent visit, basic health information (height, weight, smoking history, alcohol consumption) was collected that could be used by the practice for the National Health Service (NHS) Quality and Outcomes Framework (QOF) [10] if they wished.

Participants with a positive breath test were randomised to eradication treatment (lansoprazole 30 mg, clarithromycin 500 mg and metronidazole 400 mg twice daily for one week) or placebo. The eradication treatment and placebo were purchased in two bulk orders from MODEPHARMA [11] with expiry dates of 31st May 2014 and November 25th 2017. Medication was stored in a controlled drug storage facility set up within the Nottingham Trial Centre under the supervision and monitoring of the Nottingham University Hospitals Trust Clinical Trials Pharmacy. All randomising and posting of medication were performed by the Nottingham Trial Centre.

A bespoke HEAT web-based database and software management system was developed for the trial by TCR Nottingham [12], a company that has developed and maintain a range of software for the health community and provide support for GP practices throughout the UK. The database was housed within the secure NHS N3 Data Network and communicated directly with the HEAT Toolkit installed at the GP practices. Once a participant consented to the trial and was recorded as such on the HEAT Toolkit, basic demographic and relevant healthcare information was uploaded from the participant’s medical record to the trial database. No identifiable patient information was visible in the HEAT database, but was held securely by TCR Nottingham. Only information required for trial management was displayed.

The primary endpoint of the HEAT trial is hospitalisation due to definite or probable peptic ulcer bleeding, adjudicated by a blinded Adjudication Committee; the trial will end when 87 adjudicated primary events have occurred.

Randomised participants have been followed up by collecting information from:

- MIQUEST queries of GP practice databases, searching for clinical terms indicating a trial endpoint, as well as current relevant health and prescribing information. Participating practices were requested to perform regular searches via the HEAT Toolkit and upload the results to the HEAT web-based trial management system
- Regular requests to NHS Digital for Hospital Episode Statistics secondary care admission data [2] and mortality data from the Office of National Statistics [3], matched to the data provided by the MIQUEST searches of the GP practice records
- Event forms given to all randomised participants for the purpose of reporting any hospital admissions or changes to GP practice/home address
- Serious Adverse Event reporting by GPs. Because the trial is classified by the Medicines and Healthcare products Regulatory Agency as the lowest risk trial of an investigational medicinal product, and trial medication was only taken for one week, this was only collected for 4 weeks from the start of eradication treatment for each randomised participant

All follow-up data has been accumulated in the HEAT database from which anonymised reports can be downloaded for analysis. Success of recruitment of both GP practices and patients has been evaluated across the CRN regions of the UK. Recruitment figures were also analysed with respect to area level deprivation based on postcode. The Index of Multiple Deprivation (IMD) is a measure of relative deprivation used to rank neighbourhoods across the UK. Small areas of the country are ranked from the most deprived to the least deprived, and these are then divided into 10 equally sized groups, or deciles, numbered 1 (10% most deprived) through to 10 (10% least deprived) [13–16]. The GP practice postcode was used to determine the IMD decile for pre-consent statistics since patient domiciliary postcode was not available prior to consent being given.

## Results

### GP Practice recruitment

**Fig. 1:**
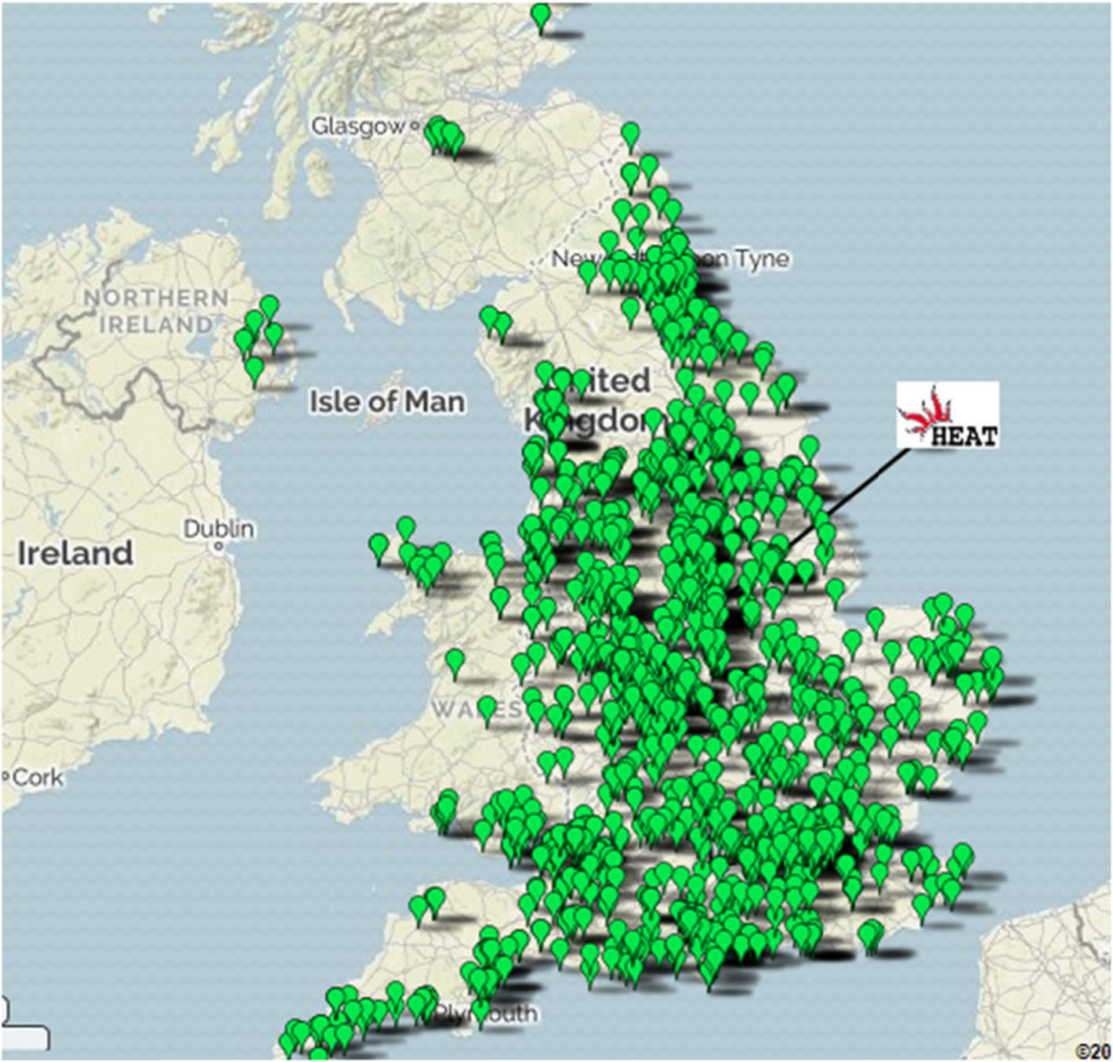
GP Practices taking part in HEAT. Each dot on the map represents individual GP practices

Practice recruitment began in 2012 and completed in 2017. Due to the large numbers of patients required, HEAT was managed from four regional centres based in Nottingham (Trial Sponsor), Southampton, Oxford/Birmingham and Durham, associated with the geographical location of the trial Principal Investigators. Each regional centre already had good contacts with the CRNs in their respective area from previous studies and was responsible for recruiting GP practices in their region. Recruitment began in the CRN regions in England closest to the regional centres but ultimately HEAT recruited from practices across the whole of the UK (Fig. 1, Table 1).

**Table 1:** GP practice recruitment in each region of the UK

UK Region	Date recruitment started in region	Total^1^ (approx.) number of GP practices in region	Number of GP practices in region recruiting to study	Practices recruiting to study as a percentage of total in region	Number of participating practices recruiting using practice nurses	Practices recruiting using practice nurses as a percentage of practices recruiting
**England**						
CRN E Midlands	14.09.2012	578	127	22.0	0	0
CRN Yorks & Humber	20.09.2012	736	119	16.2	29	24.4
CRN Wessex	27.09.2012	292	101	34.6	27	26.7
CRN Thames Valley & S Mids	05.11.2012	249	57	22.9	18	31.6
CRN SW Peninsula	21.11.2012	279	72	25.8	22	30.6
CRN Eastern	03.12.2012	431	88	20.4	60	68.2
CRN W of England	13.12.2012	281	80	28.5	30	37.5
CRN NE & N Cumbria	02.01.2013	418	65	15.6	16	24.6
CRN W Midlands	27.03.2013	886	202	22.8	6	3.0
CRN Kent, Surrey, Sussex	27.08.2013	550	63	11.5	37	58.7
CRN NW Coast	04.01.2014	619	64	10.3	36	56.3
CRN S London	28.03.2014	454	43	9.5	8	18.6
CRN N Thames	10.07.2014	837	43	5.1	3	7.0
CRN NW London	05.11.2014	388	11	2.8	6	54.5
CRN GTR Manchester	25.11.2014	502	17	3.4	4	23.5
**Total in England**		**7500**	**1152**	**15.4**	**302**	**26.2**
**Wales**						
Betsi Cadwaladr University LHB	03.02.2015	107	11	10.3	7	63.6
Cardiff and Vale University LHB	05.02.2015	66	10	15.2	10	100
Abertawe Bro Morgannwg University LHB	10.02.2015	70	6	8.6	5	83.3
Powys Teaching LHB	13.03.2015	17	4	23.5	2	50.0
Aneurin Bevan LHB	20.07.2015	80	5	6.2	3	60.0
Cwm Taf LHB	11.09.2015	42	5	11.9	4	80.0
Hywel Dda LHB	06.11.2015	51	1	2.0	0	0
**Total in Wales**		**433**	**42**	**9.7**	**31**	**73.8**
**Northern Ireland**						
Belfast HSCT	15.05.2015	82	1	1.2	0	0
Southeastern HSCT	10.11.2015	54	4	7.4	0	0
Northern HSCT	04.05.2016	75	1	1.3	0	0
**Total in Northern Ireland**		**211**	**6**	**2.8**	**0**	**0**
**Scotland**						
Tayside LHB	24.01.2017	64	3	4.7	0	0
Lanarkshire LHB	07.06.2017	104	5	4.8	0	0
**Total in Scotland**		**168**	**8**	**4.8**	**0**	**0**
**Total in UK**		**8312**	**1208**	**14.5**	**333**	**27.6**

^1^Total number of GP Practices in area obtained from:

https://digital.nhs.uk/services/organisation-data-service/data-downloads/gp-and-gp-practice-related-data (as of 31 August 2018)

https://data.england.nhs.uk/dataset/ods-northern-ireland (as of 31 August 2018)

http://www.isdscotland.org/Health-Topics/General-Practice/Workforce-and-Practice-Populations/ (as of October 2018)

Of the 195 CCGs that make up the 15 English CRNs, 169 recruited to the trial. All of the 7 Welsh LHBs recruited, 3 of the 5 Northern Irish HSCTs and 2 of the 14 Scottish LHBs recruited to the trial.

The percentage of participating GP practices in the different regions ranged from 1.2 to 34.6%. Altogether 1208 GP practices were recruited, from which a total of 188,875 invitation letters were posted to patients. Forty-six practices were enrolled into the trial but withdrew before sending out any invitation letters. Over one quarter of practices (333, 27.6%) recruited using their own practice nurses. This use varied greatly from region to region with some using no practice nurses for recruitment (eg East Midlands, Northern Ireland and Scotland where all recruitment was carried out by CRN or dedicated trial research nurses) and others with over 50% recruitment carried out by practice nurses.

### Participant recruitment

**Fig. 2:**
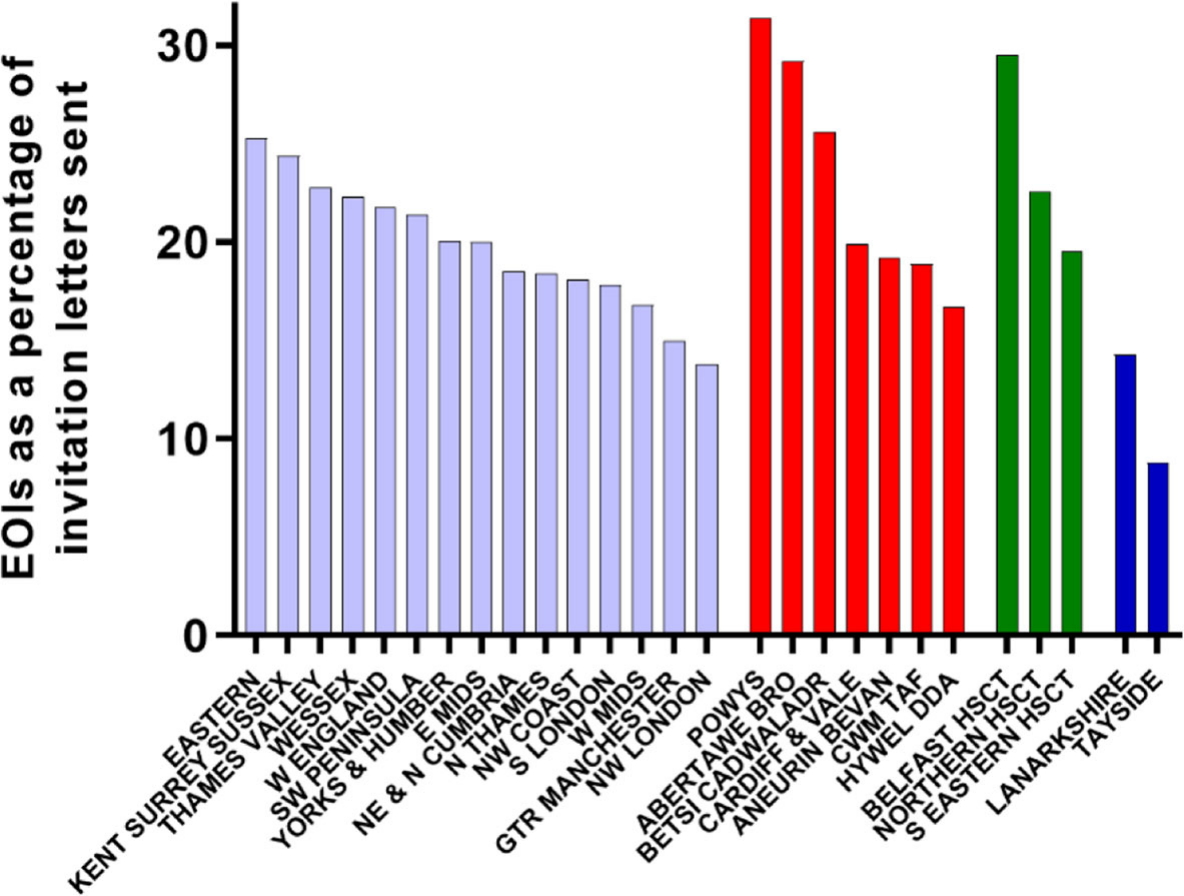
EOIs from patients invited to participate in HEAT. Bars represent recruitment in each English CRN (blue)/Welsh LHB (red)/Northern Irish HSCT (green)/Scottish LHB (dark blue) expressed as a percentage of total invitation letters sent for each region

Of the invited patients, 77,754 (41.2%) returned a reply slip (Table 2), of which 38,771 (20.5% of those invited, 49.9% of those responding) patients expressed an interest (EOI) in participating in the trial (Fig. 2).

**Table 2:** Participant recruitment in each region of the UK

Region	Total letters sent	Total reply slips received	Total expressions of interest (EOI)	EOI as a percentage of letters sent	Total^1^ consented participants	Consented participants as a percentage of EOI	Total *H. pylori*-positive participants	*H. pylori*-positive participants as a percentage of consented participants
**England**								
CRN E Midlands	20,242	8333	4051	20.0	3531	87.2	664	18.8
CRN Yorks & Humber	20,651	8306	4148	20.1	3023	72.9	606	20.0
CRN Wessex	19,070	8784	4250	22.3	3386	79.7	506	14.9
CRN Thames Valley & S Mids	9485	3946	2166	22.8	1698	78.4	249	14.7
CRN SW Peninsula	14,609	6511	3131	21.4	2631	84.0	418	15.9
CRN Eastern	14,732	6857	3728	25.3	2699	72.4	401	14.9
CRN W of England	13,248	5899	2885	21.8	2384	82.6	321	13.5
CRN NE & N Cumbria	8030	3142	1486	18.5	1154	77.7	283	24.5
CRN W Midlands	29,953	11,353	5046	16.8	4242	84.1	772	18.2
CRN Kent, Surrey, Sussex	9909	4723	2417	24.4	1653	68.4	256	15.5
CRN NW Coast	10,697	3604	1932	18.1	1312	67.9	305	23.2
CRN S London	3112	912	555	17.8	376	67.7	75	19.9
CRN N Thames	4314	1453	793	18.4	573	72.3	123	21.5
CRN NW London	974	252	134	13.8	80	59.7	26	32.5
CRN GTR Manchester	1897	592	284	15.0	222	78.2	53	23.9
**Total in England**	**180,923**	**74,667**	**37,006**	**20.5**	**28,964**	**78.3**	**5058**	**17.5**
**Wales**								
Betsi Cadwaladr University LHB	1470	620	376	25.6	229	60.9	49	21.4
Cardiff and Vale University LHB	1118	453	223	19.9	155	69.5	43	27.7
Abertawe Bro Morgannwg University LHB	1277	595	373	29.2	242	64.9	50	20.7
Powys Teaching LHB	427	214	134	31.4	83	61.9	17	20.5
Aneurin Bevan LHB	816	270	157	19.2	104	66.2	31	29.8
Cwm Taf LHB	723	283	137	18.9	102	74.5	30	29.4
Hywel Dda LHB	209	62	35	16.7	28	80.0	9	32.1
**Total in Wales**	**6040**	**2497**	**1435**	**23.8**	**943**	**65.7**	**229**	**24.3**
**Northern Ireland**								
Belfast HSCT	88	29	26	29.5	25	96.2	6	24.0
Southeastern HSCT	609	181	119	19.5	107	89.9	28	26.2
Northern HSCT	305	111	69	22.6	68	98.6	20	29.4
**Total in Northern Ireland**	**1002**	**321**	**214**	**21.4**	**200**	**93.5**	**54**	**27.0**
**Scotland**								
Tayside^2^ LHB	261	71	23	8.8	6	26.1	0	0.0
Lanarkshire LHB	649	198	93	14.3	53	57.0	23	43.4
**Total in Scotland**	**910**	**269**	**116**	**12.7**	**59**	**50.9**	**23**	**39.0**
**Total in UK**	**188,875**	**77,754**	**38,771**	**20.5**	**30,166**	**77.8**	**5364**	**17.8**

^1^A consented participant was defined as one with a valid signed Informed Consent Form and a Data Capture Record form completed at screening and entered on the HEAT database

^2^Tayside LHB withdrew from the trial shortly after starting recruitment due to staffing problems

Sixteen GP practices did not receive any patient replies even though 6 of them sent out more than 40 invitation letters (455 total letters sent), and 8 practices received only negative replies. Thirty-one GP practices did not consent any patients, despite sending out a total of 2457 invitation letters from which 632 responses were received (including 279 EOIs).

**Fig. 3:**
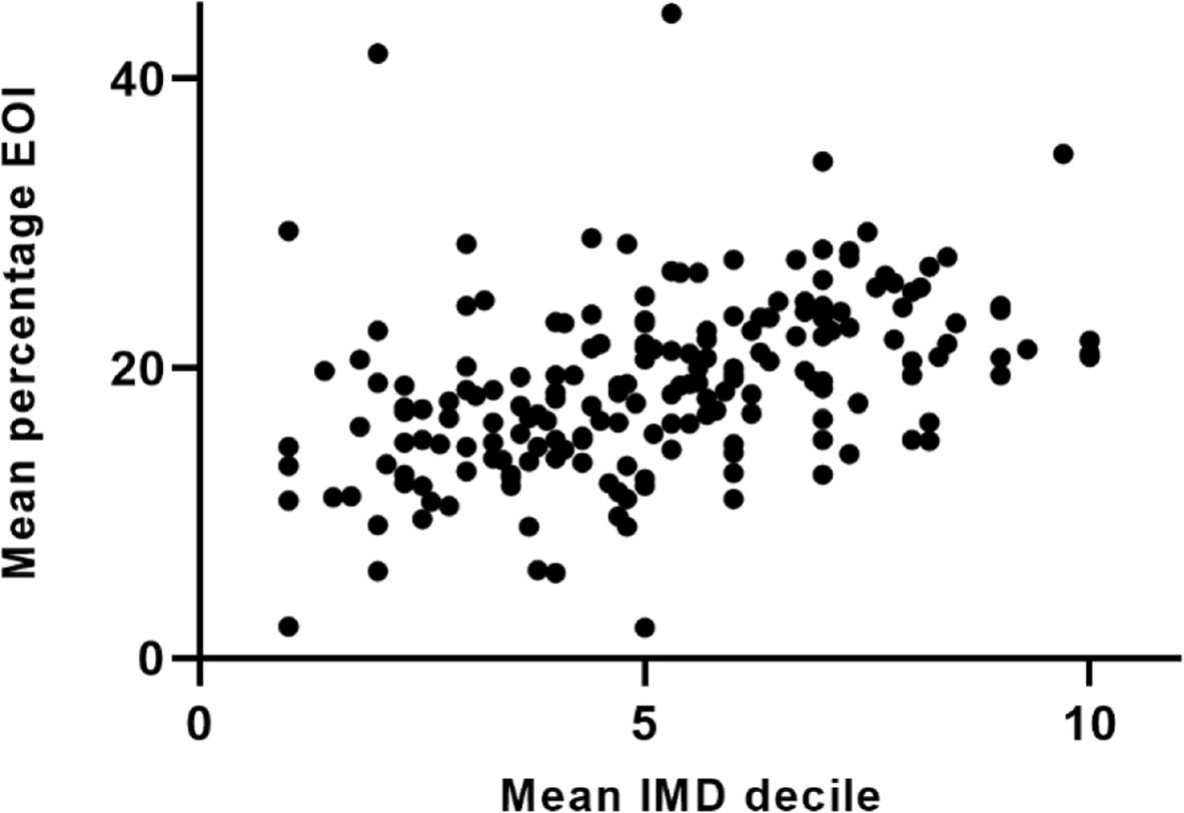
Lower expression of interest (mean percentage EOI) in deprived areas (mean IMD decile of the GP practice postcodes). For each GP practice, the EOI was calculated as a percentage of letters sent and a mean value calculated for each CCG, LHB or HSCT. These were plotted against the mean IMD decile of the practice postcode for each CCG, LHB or HSCT. Full data is shown in Appendix Table 1.

For each CCG/LHB/HSCT, the percentage of EOIs received from invited patients was analysed (Pearson correlation) against the IMD decile associated with the postcode of the GP practice and showed a moderate positive correlation (*r* = 0.42, 95%CI 0.30–0.53, *P* < 0.0001; Fig. 3, Appendix Table 1). This suggested that patients registered with GP practices situated in less deprived areas were more likely to express an interest in the trial. For the practices that failed to recruit any patients, of the 16 with no responses, 9 had postcodes in the 3 most deprived IMD deciles, and of the 31 practices with no consented participants, 18 practices had postcodes in the 3 most deprived IMD deciles.

Table 3 shows that the recruited practices were distributed fairly evenly across the 10 IMD deciles. Patients from more deprived areas were more likely to test positive for *H. pylori*, but less likely to express an interest, resulting in similar randomisation rates across all the IMD deciles.

**Table 3:** GP practice and patient recruitment with regard to IMD decile of GP postcode

	IMD decile of GP practice postcode^1^									
	1	2	3	4	5	6	7	8	9	10
Percentage of total GP practices recruited	10.5	10.6	11.0	10.8	11.1	10.1	10.2	8.5	8.4	8.7
Mean^2^ percentage of patients invited from each practice who expressed an interest	13.7 ± 6.2	15.2 ± 6.8	16.7 ± 6.6	17.9 ± 6.9	18.9 ± 7.2	21.9 ± 8.0	22.8 ± 7.7	22.5 ± 6.8	23.6 ± 6.9	23.6 ± 7.5
Mean^2^ percentage of patients invited from each practice who consented	10.1 ± 5.3	12.2 ± 6.1	13.6 ± 6.2	14.3 ± 6.8	15.2 ± 6.5	17.8 ± 6.9	18.2 ± 6.8	18.1 ± 6.5	18.5 ± 6.9	18.6 ± 6.5
Mean^2^ percentage of consented patients who were *H. pylori*-positive	26.5 ± 19.5	22.8 ± 17.4	20.5 ± 16.0	20.5 ± 16.0	20.2 ± 14.3	20.1 ± 17.6	19.4 ± 13.3	15.4 ± 9.1	15.7 ± 9.1	15.2 ± 8.5
Percentage of total randomised participants	8.7	10.1	10.5	10.6	11.7	9.8	11.4	8.2	9.2	9.8

^1^IMD deciles: 1 = most deprived, 10 = least deprived

^2^Values shown are mean ± SD

**Fig. 4:**
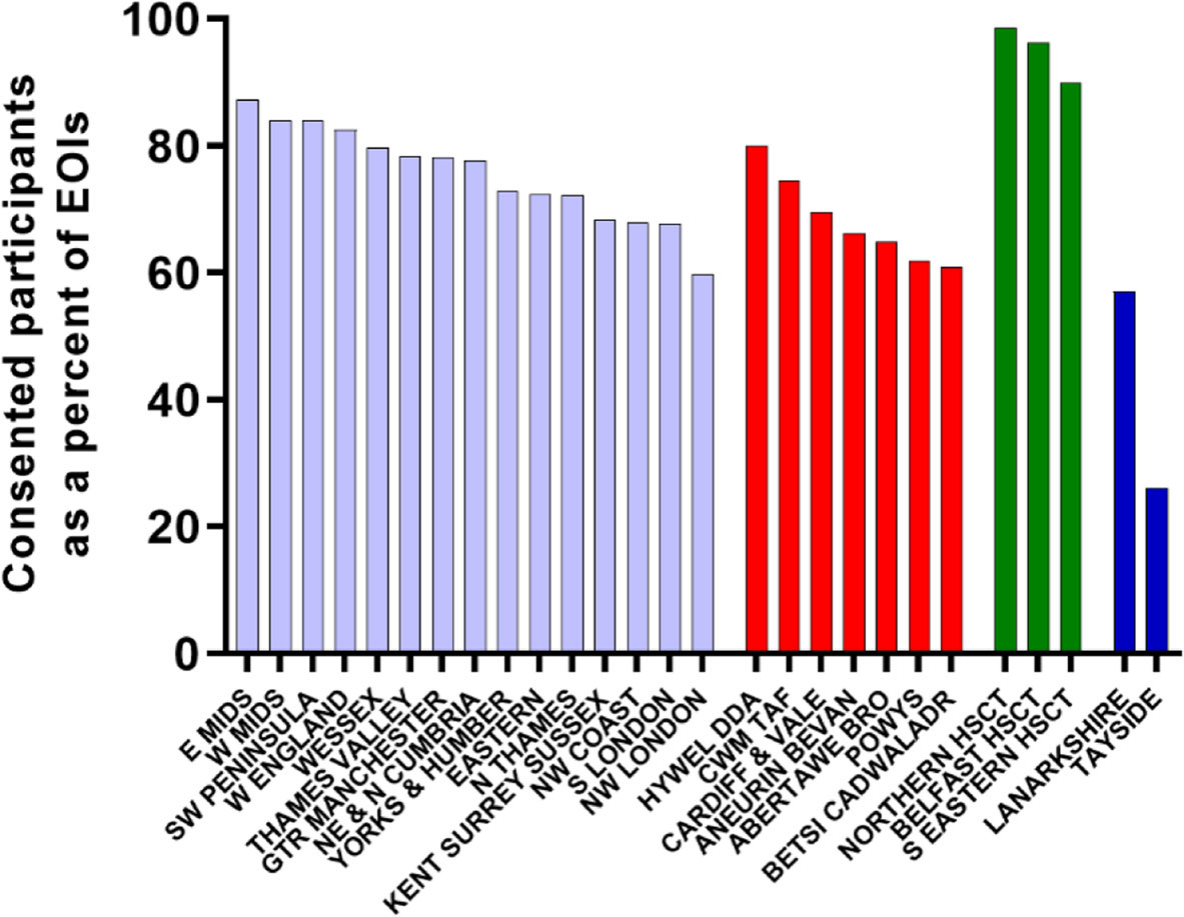
Total consented participants expressed as a percentage of total EOIs for each research network

Of the 38,771 patients expressing an interest, 31,690 attended a screening visit and 30,166 were consented (16% of those invited, 77.8% of EOI). The percentage of patients consented across the UK research networks (excluding Tayside LHB) varied between 57.0% and 98.6% of the EOIs (Fig. 4, Table 2).

Overall, 77.8% of patients expressing an interest consented to trial participation. Tayside LHB consented only 26.1% of their EOIs, none of whom went on to be randomised because of its withdrawal from the trial owing to staffing problems. All three HSCTs in Northern Ireland consented 90% or more of their interested patients.

**Fig. 5:**
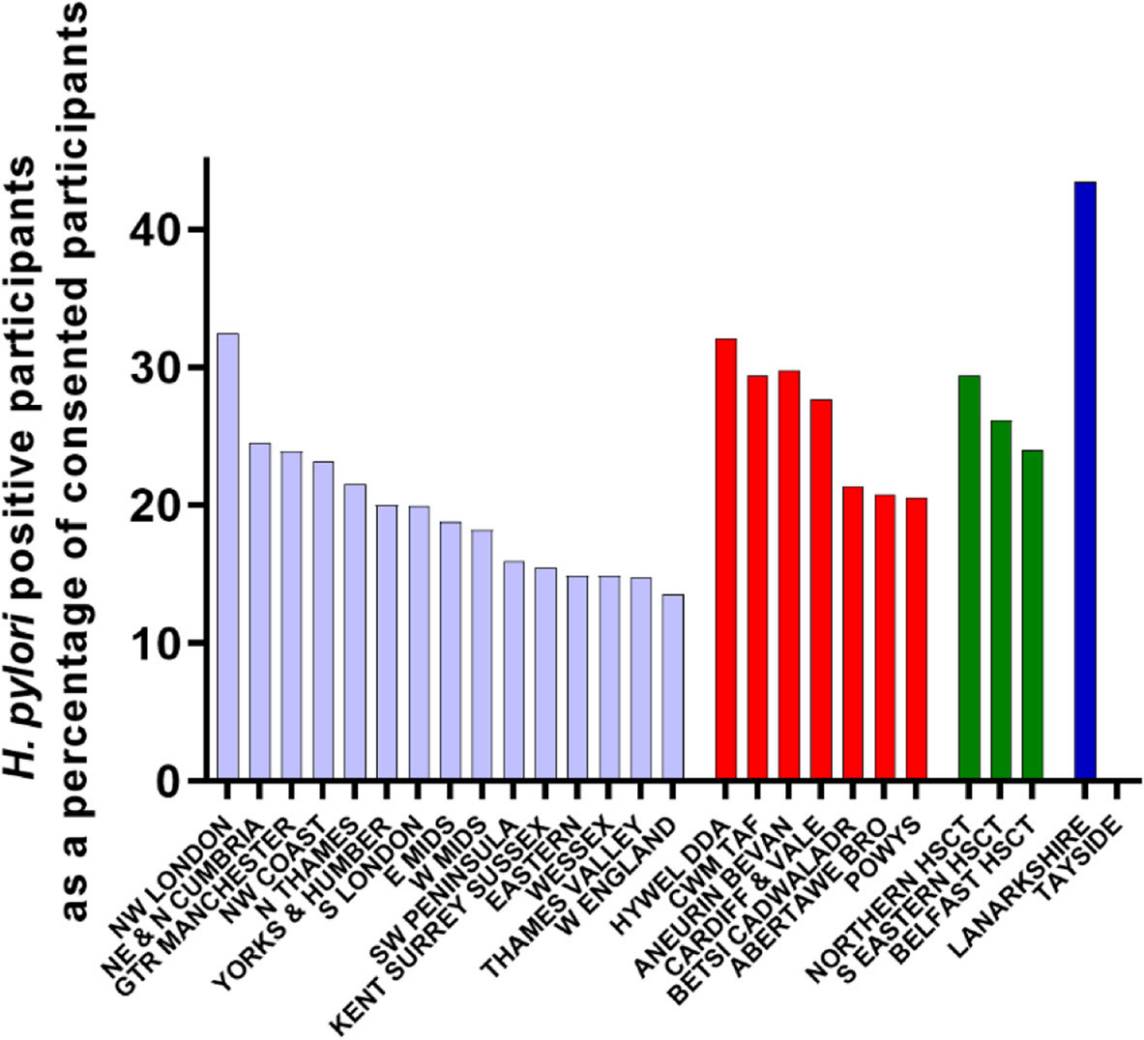
Total *H. pylori*-positive participants expressed as a percentage of total consented participants for each research network

Of the consented participants, 29,894 had a recorded breath test result of which 118 were inconclusive and 5364 positive. This represented a *H. pylori*-positive rate of 17.9%, less than the 22% rate seen in the pilot study. Of those *H. pylori*-positive participants 5355 were randomised. Across the research networks, the percentage of *H. pylori*-positive participants varied between 13.5 and 43.4% of those consented (Fig. 5; Table 2).

In England, the data suggested that *H. pylori* rates were higher in Northern regions and around London (Fig. 5). For the three devolved nations, the percentage of *H. pylori*-positive participants (24.3% in Wales, 27.0% in Northern Ireland, 39.0% in Scotland) appeared to be higher than that in England (17.5%). This difference was significant (1-way ANOVA, Sidak’s multiple comparisons test) for Wales (*p*=0.02) and Scotland (*p*=0.0004), but not for Northern Ireland (*p*=0.1)

**Fig. 6:**
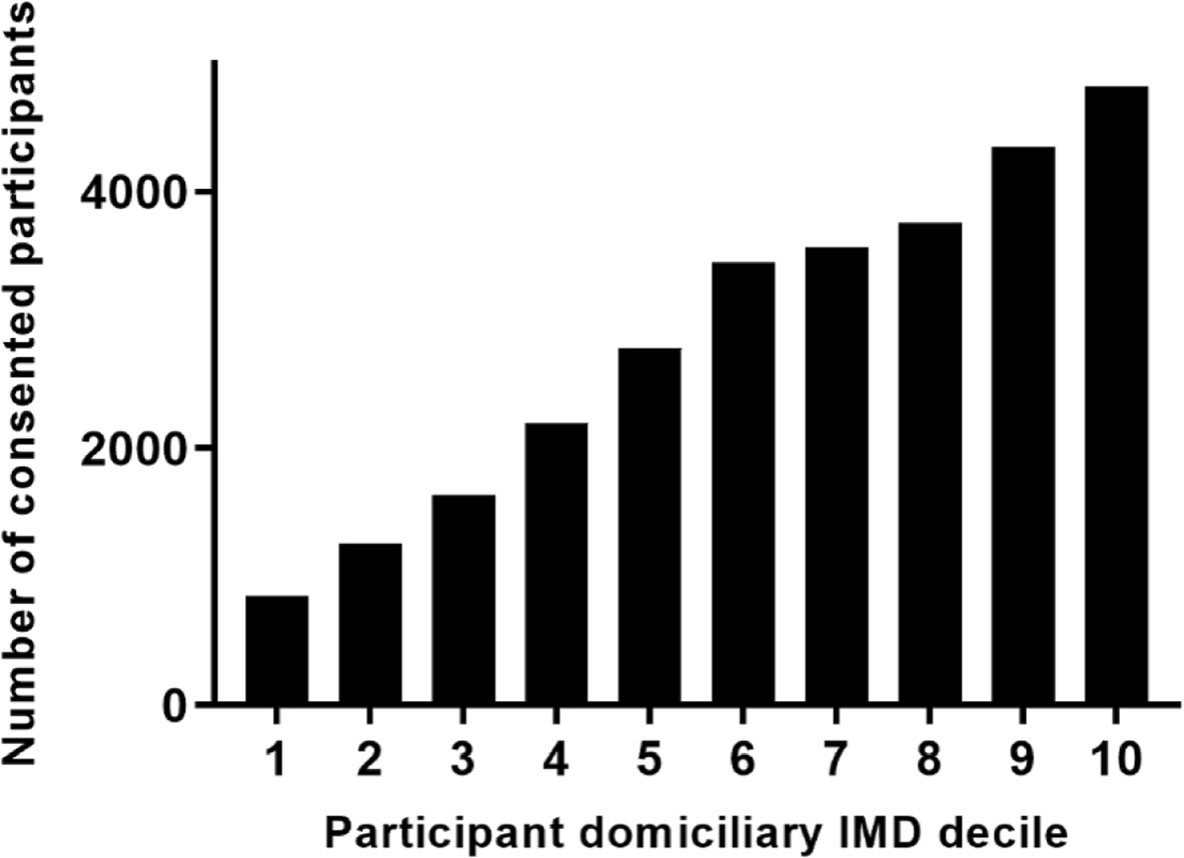
Participant consent in relation to IMD decile of their domiciliary postcode

The number of participants who consented to take part in the trial was more than 5-fold greater for those residing in areas of least deprivation (16.8% in IMD decile = 10) than those residing in areas of the greatest deprivation (3.0% in IMD decile = 1) (Fig. 6).

**Fig. 7:**
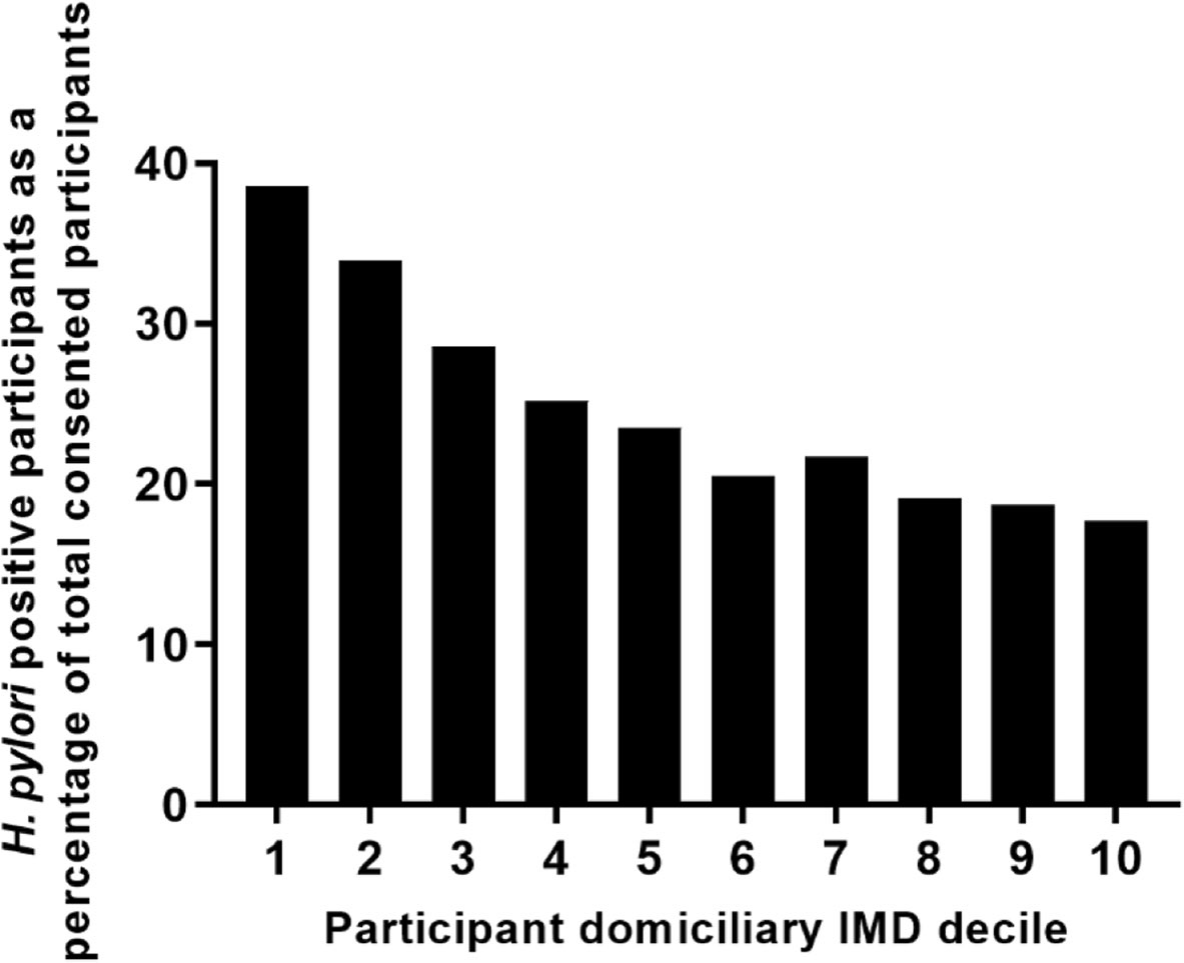
Proportion of *H. pylori*-positive participants in relation to IMD decile of their domiciliary postcode

In contrast, the proportion of those consented participants who were *H. pylori*-positive decreased as the IMD decile increased (i.e., in less deprived areas) (Fig. 7).

### Participant demographics

Of the total number of patients invited to take part in the trial, 61.5% were male (Table 4). A positive EOI was returned by 15.3% of invited females and 23.8% of invited males.

**Table 4:** Patient response to trial invitation

Patient sex	Total number of patients invited	Response to invitation			
		EOI	No	May in future	No response
Female	72,644	11,146 (15.3%)^1^	14,905 (20.5%)	1886 (2.6%)	44,707 (61.5%)
Male	116,231	27,625 (23.8%)^2^	19,274 (16.6%)	2918 (2.5%)	66,414 (57.1%)

Numbers in brackets show the percentage of total invited ^1^females or ^2^males respectively for each response to invitation

Of those patients returning an EOI, 75.1% of females and 78.9% of males completed a consent visit, and of the total consented participants, 72.2% were male and 27.8% were female.

The mean age at consent for total consented participants was 73.1 ± 6.9 (SD) years.

Only 16.8% of consented females and 18.2% of consented males returned a positive *H.pylori* breath test result (Table 5), and of the total *H. pylori*-positive participants 73.8% were male and 26.2% were female.

**Table 5:** *H. pylori* status of consented participants

Participant sex	Total number of consented participants	*H.pylori* breath test result			
		Positive	Negative	Inconclusive^1^	No result^1^
Female	8373	1407 (16.8%)^2^	6822 (81.5%)	46 (0.5%)	98 (1.2%)
Male	21,793	3957 (18.2%)^3^	17,590 (80.7%)	72 (0.3%)	174 (0.8%)

^1^Participants with an inconclusive or missing result after the initial test at consent were sent a repeat test in the post. Not all were returned for analysis and those participants with no recorded breath test result are shown as ‘No result’

Numbers in brackets show the percentage of total consented ^2^females or ^3^males respectively for H. pylori status

For those found to be *H. pylori*-positive, the mean age was 74.0 ± 7.0 (SD) years, and for those who were *H. pylori*-negative, the mean age was 72.9 ± 6.8 (SD) years.

For the *H. pylori-*positive participants, 51.9% of females and 51.5% of males were living in the 5 most deprived IMD deciles, compared with 45.5% and 43.7% of *H. pylori*-negative females and males, respectively (Table 6).

**Table 6:** *H. pylori* status of consented participants by domiciliary IMD decile

		Number of participants							
	*H. pylori* status	Positive		Negative		Inconclusive		No result	
	IMD^1^ decile	1–5	6–10	1–5	6–10	1–5	6–10	1–5	6–10
Participant sex	Female	731 (8.7%)^2^	676 (8.1%)	3107 (37.1%)	3715 (44.4%)	25 (0.3%)	21 (0.2%)	55 (0.7%)	43 (0.5%)
	Male	2037 (9.3%)^3^	1920 (8.8%)	7695 (35.3%)	9895 (45.4%)	35 (0.2%)	37 (0.2%	96 (0.4%)	78 (0.4%)

^1^IMD deciles: 1 = most deprived, 10 = least deprived

Numbers in brackets show the percentage of total number of consented ^2^females (8373) or ^3^males (21793) respectively for *H. pylori* status according to domiciliary IMD decile

### Randomised participant withdrawals

Of the randomised (*H. pylori*-positive) participants, 73.5% remain in the trial with active contact and data follow-up as of 17 Sep 2020. Most patients returned a confirmation that they had received and taken trial treatment, but 4.18% did not and were assumed not to have taken the treatment. Reasons for early and late withdrawal are shown in Table 7.

**Table 7:** Randomised participant withdrawals (as of 17 Sep 2020)

Reason for withdrawal	Number of participants	Percentage of total randomised participants
**Treatment phase**		
Treatment sent but no response from the patient	224	4.18
Incorrectly enrolled in the trial	17	0.32
Adverse reaction to trial treatment	55	1.03
Did not want to take medication/risk side effects	18	0.34
**Follow-up**		
Consent to active contact and use of electronic data withdrawn	73	1.36
Consent to active follow-up contact withdrawn; continuing use of electronic data allowed	457	8.52
Participant died or terminally ill	531	9.92
Withdrawn at request of GP	37	0.69
Withdrawal by the participant for health reasons	5	0.09

As of September 17 2020, 4395 (82.1%) of randomised participants remain under electronic follow-up, including those who withdrew consent to active contact but allowed continuing collection of their electronic data.

## Discussion

The HEAT trial has demonstrated that large numbers of patients can be recruited into a clinical trial solely from primary care, by simplifying and streamlining the trial processes and minimising the workload for GP practices.

Over 1200 GP practices from across the UK engaged with the trial, some of which had never taken part in research before. Just under 29,000 patients were recruited in England over a period of 3 years, with another 1200 added when recruitment was extended to the devolved nations in 2015.

The prevalence of *H. pylori* was lower than expected, requiring expansion of the trial into the devolved nations, but recruitment in these nations was limited by delays in set-up and the expiry date of the eradication medication. Despite this, 91% of the target number of consented participants was achieved and 80% of the target number of randomised participants.

The UKCRN played a large role in facilitating HEAT. The figures shown in Tables 1 and 2 demonstrate that high recruitment numbers are possible with the assistance of the research networks, enabling recruitment to take place across the whole of the UK whilst managing the trial from a few coordinating centres. In addition to consenting patients, the CRN have a network of research active GP practices that they can approach to take part in clinical trials and many of them also facilitate trial training and the running of the trial at the practice.

One of the main objectives of the trial has been to develop a methodology that would enable GP practices to take part with a minimal workload burden on the practice [17, 18]. To that end, several academic GPs were members of the Trial Management Group that developed the trial protocol and procedures. Under their guidance, various processes were set up to make the practice’s role as simple as possible.

- GPs taking part in the study were given the position of Study Site Coordinators rather than Principal Investigators so that the burden of obtaining all regulatory approvals fell to the trial team rather than the practice
- The trial offered study-specific Good Clinical Practice training to non-consenting staff covering points specific to their role in the trial
- No targets for recruitment were set
- Practices were provided with a thorough electronic search tool that produced a list of eligible patients requiring minimal checking by the SSC
- All invitation letters were sent by a secure electronic mailing system relieving work load on practice administrative staff
- All consent was performed by trained research or practice nurses

The percentage of GP practices taking part in the trial varied greatly across the regions, but this was constrained in some areas, notably in Scotland, by local resource (e.g., CRN nurse availability for consenting) or budget restrictions, such as CRN-provided Service Support Costs for GP practices. Some regions experienced delayed recruitment due to IT issues; principally due to the presence of local firewalls preventing the installation of the HEAT Toolkit at GP practices. In some instances, resolution was achieved only after long discussions between local IT teams and the designers of the HEAT Toolkit (TCR Nottingham Ltd.).

The recent introduction of the General Data Protection Regulation (GDPR) [19] has increased sensitivity to installation of external software onto GP practice computers and external data transfer, as well as affecting the collection of follow-up data from NHS Digital and the Office of National Statistics, making the application process more complex. The design of a large-scale clinical trial such as HEAT depends heavily on electronic methods of data collection both for its results and for time- and cost-saving, and future trials could be severely hindered if such data were not readily available.

Collection of follow-up data can be challenging when a trial runs over several years. Many of the HEAT GP practices failed to perform the data uploads at regular intervals during the trial period and have had to be chased for final uploads at the end of the trial. This is inevitable once the recruitment period is over, and the trial is no longer uppermost in the minds of practice staff. Many practices change staff members, get new computers, or change their clinical system, often resulting in the removal of the HEAT Toolkit from their computers. Such problems can be overcome by regular automated reminders to practices to perform these uploads, and this also has the advantage of keeping the practice aware that the trial is still running.

Since the HEAT trial started, NHS Digital have changed the coding vocabulary used for searching clinical systems, implemented in GP clinical systems from April 2018. Although MIQUEST is still functional, it may no longer be developed to fully use the new coding vocabulary after the changeover [20]. However, an alternative process using automated data extraction is available in the two GP clinical systems used by the majority of practices (EMIS and TPP SystmOne), and this is most likely the way forward for future trials. Using such a process would also remove the burden on the practice to perform regular data uploads and would ensure constant, up-to-date follow-up information for the trial.

For the patients, participation in the trial was also made as convenient as possible. Only one appointment at their local GP practice was required and travel costs were reimbursed on request. Trial medication was posted to the participant’s home and pre-paid envelopes were provided for the return of any trial documents. Members of patient participation groups were incorporated in the Trial Management Group to advise on wording of patient-facing documents and consent procedures, and to ensure that the trial was as patient-friendly as possible.

Despite these measures, overall recruitment figures (30,166 patients consented, 5355 randomised) were less than the target figures of 33,000 patients consented and 6600 randomised. Recruitment was halted in October 2017 due to expiry of the eradication treatment and prohibitive costs of supplying further medication. Nevertheless, at this point, the target posting of invitation letters had been exceeded, whilst 91% of the consented participant target had been achieved and 81% of the randomised participant target.

The *H. pylori*-positive rate was lower than that seen in the pilot study, on which the original target participant numbers were based. This prompted expansion of the trial into the devolved nations, and in fact, these regions did appear to have higher rates of *H pylori*-positive patients (Fig. 5). Unfortunately, these regions did not start recruiting into the trial until it had already been up and running for 2 years, and represented a missed opportunity to increase the numbers of randomised participants. For future trials such as HEAT recruiting large numbers of patients, it may be beneficial to explore whether the clinical condition under investigation has any geographical distribution pattern and carry out preliminary test searches if possible in potential recruitment areas.

Similarly to that seen by Vyse et.al. (2002) [5], our data showed that *H. pylori* was more prevalent in the North and the areas around London. Social deprivation appears to be a major factor in prevalence of *H. pylori* [21, 22], and our data supported this, based on both participant domiciliary postcodes (Fig. 7) and GP practice location (Table 3). GP practice postcodes are not generally used for analysis as they cannot give such an accurate representation as domiciliary postcodes (as indicated by the high variability (Table 3)). Some practices can sit between the borders of postcode regions with quite different IMD deciles and are likely to have a more variable patient population. Nevertheless, using GP practice postcodes also showed an increased patient volunteering rate in practices located in less deprived areas.

Of the HEAT consented participants, 70% resided in areas of least deprivation (IMD decile 6–10, Fig. 7), and this is likely to have contributed to the lower rate of *H. pylori* infection observed in the trial. Strategies for encouraging trial participation in more socially deprived areas have been considered in other studies, including community-based recruitment [23]. Jennings et. al. (2015) [24] offered an incentive payment to encourage participation in five clinical trials in Scotland but found that it did not attract more patients from socially deprived areas. A more in-depth analysis of effect of IMD decile on trial participation in HEAT will be carried out at a later date once all data have been finalised.

Of the 38,771 patients expressing an interest in the trial, there were over 7000 who did not attend a consent clinic. There could have been several reasons for this. Patients may have changed their mind, or other events may have intervened in the period between expressing an interest and being contacted to attend a consent clinic. For some practices, there was a significant delay between inviting patients and setting up the clinics, oftentimes due to lack of availability of clinic rooms. Likewise, the consent clinics were scheduled during daytime working hours and although eligible participants were aged 60 or over, some were in full-time employment and evening clinics might have been more convenient.

Some of the larger practices had a very high response rate and the CRN nurses who work across multiple studies may not have had capacity to see all of the patients. Similarly, practice nurses consenting patients also have many other demands on their time, and research can be a lower priority. GP practices have had increasing demands on their workloads over the past few years unmatched by increases in funding or workforce [25], but despite this over 1200 took part in the HEAT trial, and over a quarter recruited using their own practice nurses. At present, GP practices are compensated by the research networks for their time but have no monetary benefit from taking part in research. Perhaps if they were rewarded for participating in research, for example by utilising QOF [10], or providing funding for research time, more practices could get involved.

The strategy for GP practice recruitment in different CRN regions varied. Some recruited a lot of practices in a short time period, whereas others staggered practice recruitment to match nurse (and financial) capacity. In these regions fewer practices were recruited, but the percentage of consented patients relative to the number of EOIs was greater.

The number of patients expressing an interest in the trial represented a 20.5% volunteering rate, which was less than that seen in the pilot study (37% volunteering rate). This may have been due to the presence of a placebo. All of the participants in the pilot study found to be positive for *H. pylori* were treated with eradication therapy, whereas participants in the trial were blinded to the treatment they received. Participants who withdrew and returned their tablets post randomisation generally gave a reason related to size and number of tablets or concern about side-effects, but some also stated that they would prefer to get treatment from their GP rather than be given a placebo, despite the risk-benefit discussion during their consent visit. The target age group for the pilot study was also different and was open to patients aged 45 or over. For a small proportion of patients the pilot study might have offered the opportunity of eradication with no prescribing costs, and encouraged participation.

With such a large trial recruiting older participants, it is inevitable that some were lost to follow-up through death (9.9% of randomised participants). HEAT did not require follow-up visits and used routinely collected electronic clinical data. Consequently, although 1417 participants were recorded as withdrawals, full continuing data collection was possible in all but the 84 randomised participants (1.57%) who actively withdrew their consent to all follow-up.

Many of the participants who withdrew without specifying a reason (457 who declined further active contact, but consented for continued electronic follow-up (Table 7)) did so in response to the annual letter sent out to randomised participants and the letter sent out to explain GDPR. The annual letters gave participants an update on trial progress, but also contained text reminding them that they were free to withdraw from the trial at any time. Participants in the HEAT trial attended for only one visit, took medication for only one week, and were subsequently followed up electronically with no personal contact, and hence may have forgotten that they were taking part in a trial. Trial participation is voluntary and a very important part of informed consent is the freedom to withdraw at any time. In the development of trial correspondence it may be beneficial that all letters, both invitation and follow-up, are reassuring to the participant in terms of current and future commitment, but also encourage and maintain interest in the trial outcomes.

## Conclusion

The HEAT trial has provided much useful information for the design and planning of future trials of this size and many lessons have been learnt. With a large study involving many practices and personnel it can be difficult to keep oversight of individual recruitment sites. Recruiting GP practices to maintain pace with capacity, completing recruitment at one practice before starting too many new ones, and making clinic times more flexible could contribute to better recruitment for future studies.

Nevertheless, this large ongoing trial has developed methodology showing that recruitment of large numbers of patients from primary care is attainable and could be used in other clinical studies.

## Supplementary Information

**Additional file 1.** Appendix Table 1.

## Data Availability

The datasets used and/or analysed during the current study are available from the corresponding author on reasonable request.

## Abbreviations

ANOVA: Analysis of variance
CCG: Clinical Commissioning Group
CI: Confidence interval
CRN: Clinical research network
EOI: Expression of interest
GDPR: General Data Protection Regulation
GP: General Practitioner
*H. pylori*: *Helicobacter pylori*
HEAT: Helicobacter Eradication Aspirin Trial
HSCT: Health & Social Care Trusts
IMD: Index of Multiple Deprivation
IT: Information Technology
LHB: Local Health Board
MIQUEST: Morbidity Information Query and Export Syntax
NHS: National Health Service
NIHR: National Institute of Health Research
QOF: Quality & Outcomes Framework
SD: Standard deviation
SSC: Study Site Coordinator
UK: United Kingdom
UKCRN: United Kingdom Clinical Research Network
